# MULTISECTOR COLLABORATION TO PROMOTE HEALTHY AGING: A PRACTICAL GUIDE FOR PUBLIC HEALTH

**DOI:** 10.1093/geroni/igae098.2512

**Published:** 2024-12-31

**Authors:** Meghan O’Leary, Catharine Fromknecht, Yen Lin, Kathryn Costello

**Affiliations:** NORC at the University of Chicago, Chicago, Illinois, United States; NORC at the University of Chicago, Chicago, Illinois, United States; US Department of Health and Human Services, Washington, District of Columbia, United States; US Department of Health and Human Services, Washington, District of Columbia, United States

## Abstract

In collaboration with the HHS Office of Disease Prevention and Health Promotion, NORC at the University of Chicago developed a Quick Start Guide designed for public health officials to start or enhance partnerships with state officials who work with the older adult population. As the proportion of Americans over the age of 65 continues to increase, multisector partnerships will be critical to address the needs of this population and ensure that older adults live healthy, happy lives. The Quick Start Guide includes a set of recommendations for public health departments to use as a starting place for engaging with their counterparts in state and local agencies that are focused on healthy aging. The Quick Start Guide was developed based on interviews with state health department staff and is designed to be used by public health departments at any stage in the process of collaboration, either just beginning or building on existing collaboration. This session will summarize the recommendations in the Quick Start Guide and share examples of how state health departments have successfully partnered with their counterparts at state aging agencies.

